# A simplified protocol for the detection of blood, saliva, and semen from a single biological trace using immunochromatographic tests

**DOI:** 10.1007/s12024-021-00453-2

**Published:** 2022-02-16

**Authors:** Patrick Basset, Prisca Blandin, Annalisa Grini, Séverine Delemont, Lydie Samie, Vincent Castella

**Affiliations:** Forensic Genetics Unit, University Centre of Legal Medicine Lausanne-Geneva, Lausanne University Hospital and University of Lausanne, Chemin de la Vulliette 4, Lausanne 25, CH-1000 Switzerland

**Keywords:** Body fluid, Immunochromatographic tests, OBTI, PSA or RSID tests

## Abstract

The detection of body fluids (e.g., blood, saliva or semen) provides information that is important both for the investigation and for the choice of the analytical protocols. Because of their sensitivity, specificity, as well as their simplicity of use, immunochromatographic tests are widely applied. These tests target different body fluids and generally require specific buffer solutions. If one needs to investigate whether the material is of a specific nature (e.g., blood), this is fine. However, if the material can also contain other material (e.g., saliva or semen) then the use of different tests can be problematic. Indeed, if the different tests require different buffers, it will not be possible to perform all tests on the exact same specimen.

In this study, we assess the use of the RSID™-universal buffer to perform three immunochromatographic tests (HEXAGON OBTI, RSID-saliva, and PSA Semiquant) as well as spermatozoa detection. We use the same eluate for the detection of all three body fluids. The proposed protocol provides similar results to those obtained when each test is conducted independently. Furthermore, it does not affect the quality of the DNA profiles. The main advantage of this protocol is that the results of the presumptive test(s) and of the DNA analyses are representative of the exact same specimen.

**Supplementary Information:**

The online version contains supplementary material available at 10.1007/s12024-021-00453-2.

## Introduction

Body fluids such as blood, saliva, or semen are often found at crime scenes [1]. Their detection is of major importance both for the investigation and for the examination strategy. Immunochromatography, spectroscopy, RNA expression, or methylation patterns [1–3] provide information on the nature of body fluids. In particular, several commercial immunochromatographic assays are available to test for the presence of blood [4, 5], saliva [6] and semen [7, 8]. Because of their high sensitivity, specificity, speed, low cost, as well as their simplicity of use [2], they are commonly applied by law enforcement agencies.

Different strategies are available for investigating the presence of a specific body fluid in a biological trace that needs to undergo DNA analysis. One can sample the trace, use part of it for the presumptive test, and keep the remaining material for DNA analyses. Alternatively, one can take several samples from the original trace and use them separately for the presumptive test(s) and the DNA analyses. The main disadvantage of this sampling strategy is that if the material is heterogeneous, the result obtained will not be representative of the whole trace. The elution of all the material in a single buffer allows performing both the presumptive test(s) and the DNA analysis on the same specimen. However, a careful optimization is required to find a good trade-off between the optimization of the detection of body fluids and of DNA analysis.

Commercial immunochromatographic tests targeting different body fluids differ in their sensitivity and in their technical characteristics. These tests generally require specific buffers for the elution and the migration of the biological material. This prevents the use of different tests on the same eluted trace. Exceptions exist such as the combination of the RSID-Semen & RSID-saliva tests validated by the provider (https://www.ifi-test.com/rsid-saliva/) or the combination of strip tests for the simultaneous detection of five body fluids [9]. Yet, this latter method was a proof of principle and no commercial test is currently available.

The aim of this study was to develop a protocol that allowed performing all three immunochromatographic tests with the same buffer, as we wanted to provide answers for the presence of blood, saliva, and semen in the same specimen. It was also important that the protocol allowed the detection of spermatozoa by microscopy, as this is a more specific examination. Based on the characteristics of the tests and our experience with those tests in caseworks, we chose the HEXAGON OBTI (hereafter OBTI) test by Human (Wiesbaden, Germany) for the detection of blood. This test, which targets human hemoglobin, has a high sensitivity and has been used for years within the medical and forensic fields [4]. For saliva detection, we selected the RSID-saliva test (Independent Forensics, Hillside, USA), which targets human alpha-amylase, an enzyme abundant in human saliva [6]. Finally, for semen detection, we adopted PSA Semiquant (hereafter PSA) test by SERATEC® GmbH (Goettingen, Germany). This test targets human prostate-specific antigen (PSA) which is a glycoprotein produced by prostatic epithelial cells. Although PSA can sometimes be present in low concentration in body fluids other than semen, such as urine or breast milk, it has a high sensitivity, and it is commonly used to look for semen in forensic caseworks [8, 10]. A high sensitivity was requested within our laboratory, since we look for the presence of sperm cells using Christmas tree staining (hereafter Christmas Tree [11]) only when the presumptive test for the presence of semen is positive. Consequently, the proposed protocol should also be compatible with the detection of sperm cells using microscopy.

## Material and methods

### Description of the protocol

**Fig. 1:**
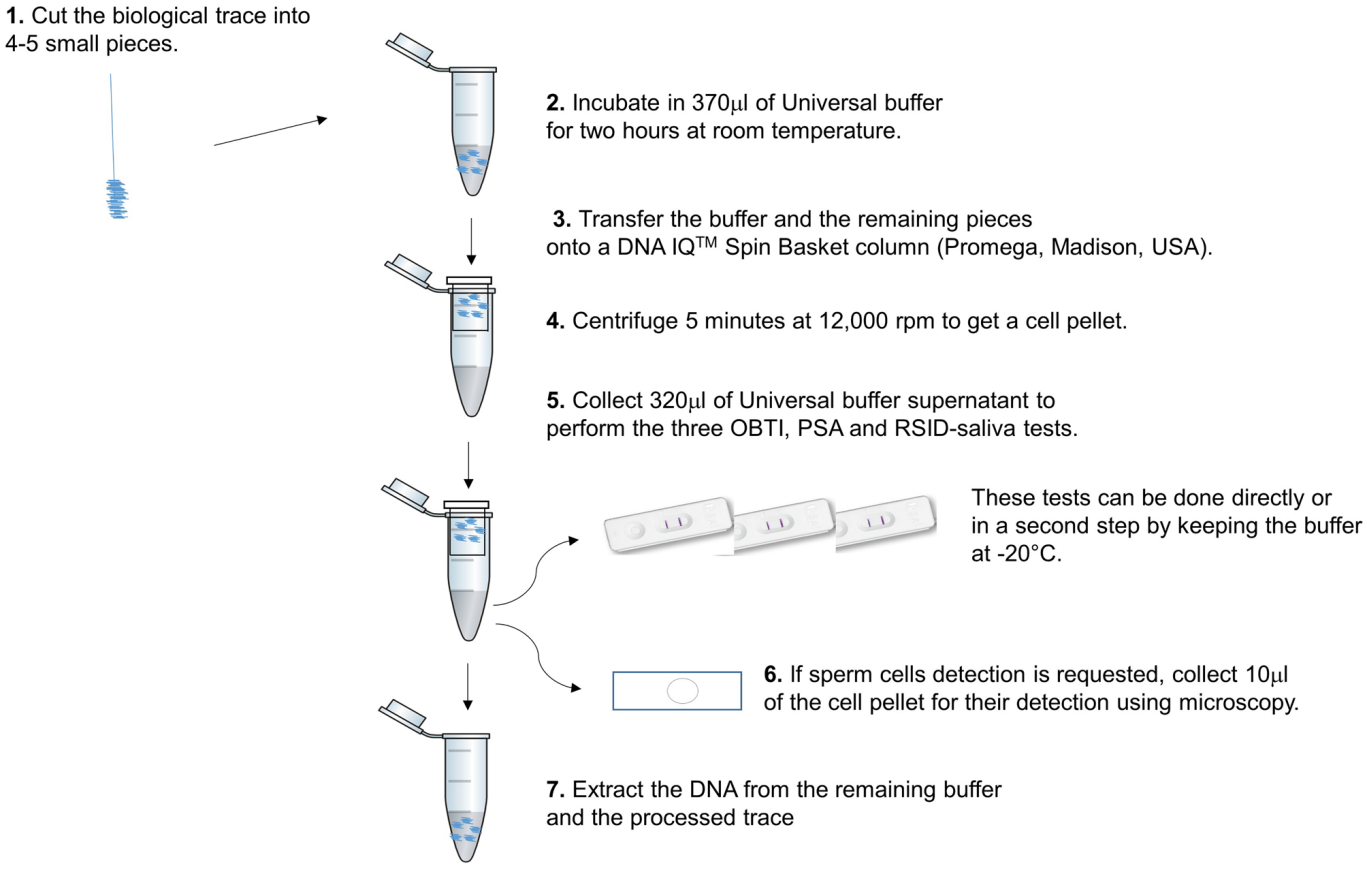
Detailed steps of the proposed protocol

The protocol proposed here allows performing three presumptive tests plus sperm cell detection from a single biological trace. These tests can take place simultaneously or at different moments. Our protocol was optimized and tested using 4N6 FLOQSwabs™ from Copan (Italy). For the three immunochromatographic tests used in this study (OBTI, PSA, and RSID-saliva), the trace was first eluted using a buffer solution which was then deposited on the selected test strip. The three tests considered here are from different suppliers and come with different buffers: OBTI buffer for the OBTI test, PSA buffer for the PSA test, and RSID™-universal buffer (hereafter referred as universal buffer) for the RSID-saliva test. Our aim was to use a single buffer for all three tests. According to preliminary results (not shown), we chose to incubate the biological trace into 370 μl of universal buffer and to collect the amount of buffer supernatant necessary to perform the three OBTI, PSA, and RSID-saliva tests. We describe the different steps of our protocol in Fig. 1.

### OBTI or PSA buffer vs. universal buffer

The manufacturers recommend to elute the trace and perform the OBTI or PSA with their respective buffers [14, 15]. Our new protocol replaces them with the universal buffer. To ensure that this change had no major impact on the results, we analyzed the same specimens with the recommended buffers and with the universal buffer. We collected blood from one volunteer and prepared eleven dilutions of this blood at the same time (see Table 1 for the description of the dilutions). Another volunteer gave a sperm ejaculate from which five dilutions were prepared (see Table 2 for the description of the dilutions). For each fluid, 20 µl of these dilutions were deposited on 4N6 FLOQSwabs™. Before the deposition of semen dilutions, an analyst collected buccal cells from the mouth of a female volunteer with 4N6 FLOQSwabs™. This allowed having a mixture of epithelial cells and semen. For each dilution, we analyzed three swabs using the ad hoc and universal buffer (see protocol described in Fig. 1). To follow our examination strategy for PSA positive tests, we performed microscopy after Christmas Tree staining [11]. This was done using 10 μl of the buffer (see step 6 of Fig. 1) for both buffers and for each dilution. The whole slide was observed with a magnification of 500 × .

**Table 1:** Comparison of the results of the OBTI test (OBTI) and mean DNA quantity recovered from the three swabs ± standard deviations [ng] (DNA quantity), according to dilutions after elution with the OBTI or universal buffer. The proportion of DNA recovered in the buffer compared to the DNA recovered from the swab is indicated for the universal buffer only as well as the mean number of alleles of the donor (out of 28 donor alleles) called from the STR-profile (Nb alleles)

Blood dilutions	OBTI buffer		Universal buffer				
	OBTI^1^	Mean DNA quantity	OBTI^1^	Mean DNA quantity	Proportion DNA in buffer		Nb alleles^2^
1	+ , + , +	312.86 ± 21.11	+ , + , +	189.98 ± 23.30		0.8 ± 0.2%	NT
1/20	+ , + , +	21.38 ± 2.31	+ , + , +	18.71 ± 2.10		1.4 ± 0.1%	NT
1/80	+ , + , +	6.90 ± 1.55	+ , + , +	6.04 ± 0.83		0.3 ± 0.1%	NT
1/200	+ , + , +	2.41 ± 0.16	+ , + , +	2.41 ± 0.18		0.1 ± 0.1%	NT
1/400	+ , + , +	1.22 ± 0.16	+ , + , +	1.28 ± 0.36		0.3 ± 0.2%	NT
1/800	+ , + , +	0.49 ± 0.09	+ , + , +	0.74 ± 0.24		0.3 ± 0.3%	27.7 ± 0.6
1/2′000	-,-,-	0.26 ± 0.10	-,-,-	0.25 ± 0.06		0.7 ± 1.6%	25.0 ± 0.0
1/4′000	-,-,-	0.07 ± 0.01	-,-,-	0.09 ± 0.02		0.0 ± 0.0%	22.7 ± 2.3
1/8′000	-,-,-	0.05 ± 0.01	-,-,-	0.02 ± 0.01		0.0 ± 0.0%	8.7 ± 4.2
1/12′000	-,-,-	0.01 ± 0.01	-,-,-	0.01 ± 0.01		0.0 ± 0.0%	6.0 ± 2.0
1/20′000	-,-,-	0.01 ± 0.01	-,-,-	0.01 ± 0.01		0.0 ± 0.0%	2.0 ± 2.0

^1^ − Negative, + positive results,

^2^*NT* not tested

**Table 2:** Comparison of the results for the Christmas analysis (Christ.), PSA test (PSA), and mean DNA quantity recovered from the three swabs ± SD [ng] in the epithelial (DNA quant. epith.) and sperm (DNA quant. sperm.) fractions, according to the sperm dilutions tested after elution with the PSA and universal buffers. The proportion of sperm DNA recovered in the buffer and the mean number of reference alleles called from the 31 sperm reference alleles profile (nb alleles) is indicated for the universal buffer

Sperm dilutions	PSA buffer				Universal buffer					
	Christ.^1^	PSA^2^	DNA quant. epith	DNA quant. sperm	Christ.^1^	PSA^2^	DNA quant. epith	DNA quant. sperm	Proportion of sperm DNA in buffer	Nb allele^3^
1/50	+ , + , +	+ , + , +	0.83 ± 0.47	59.05 ± 16.31	+ , + , +	+ , + , +	5.86 ± 8.805	38.69 ± 10.38	0.0 ± 0.0%	NT
1/200	+ , + , +	+ , + , +	12.16 ± 19.61	11.25 ± 3.90	+ , + , +	+ , + , +	5.64 ± 8.870	10.43 ± 0.46	0.0 ± 0.1%	NT
1/3′200	1,1,4	+ ,( +), +	4.92 ± 8.47	0.05 ± 0.02	2,NA,8	+ , + , +	2.07 ± 3.235	0.08 ± 0.07	1.1 ± 1.9%	10.5 ± 0.7
1/5′000	1,1,4	+ , + ,( +)	7.86 ± 13.15	0.22 ± 0.29	1,1,3	+ , + , +	0.54 ± 0.530	0.03 ± 0.01	0.0 ± 0.0%	23.5 ± 0.7
1/10′000	0,0,0	-,-,-	13.09 ± 22.61	0.00 ± 0.01	0,0,0	-,-,-	1.05 ± 1.570	0.00 ± 0.00	0.0 ± 0.0%	6.5 ± 9.2

*NA* not applicable

^1^ + More than 10 sperm cells

^2^ − Negative, ( +) weak positive, + positive results

^3^*NT* not tested

As the universal buffer is recommended to elute the trace before performing the RSID-saliva test [16], the use of this buffer should not affect its performances. Nevertheless, to assess the results of this test, 20 µl of five dilutions of saliva self-collected from one female volunteer (see Table 3 for the description of the dilutions) were deposited on 4N6 FLOQSwabs™. For each dilution, we analyzed three swabs according to the protocol described in Fig. 1.

**Table 3:** Results of the RSID-saliva test (RSID), mean DNA quantity recovered from the three swabs ± standard deviations [ng] (DNA quant.), proportion of DNA recovered in the buffer as well as the mean number of reference alleles called from the 28 reference alleles profile (nb alleles), according to saliva dilutions after elution with the universal buffer

Saliva dilutions	Universal buffer			
	RSID^1^	DNA quant	Proportion DNA in buffer	Nb alleles^2^
1/50	+ , + , +	0.41 ± 0.08	21.8 ± 1.2%	NT
1/100	+ , + , +	0.16 ± 0.01	26.8 ± 3.1%	26.7 ± 1.2
1/200	( +),( +),( +)	0.37 ± 0.07	14.9 ± 3.2%	26.7 ± 1.2
1/400	-,-,-	0.14 ± 0.06	16.3 ± 4.4%	27.0 ± 1.0
1/800	-,-,-	0.05 ± 0.01	20.6 ± 4.2%	21.3 ± 2.5

^1^ − Negative, ( +) weak positive, + positive results

^2^*NT* not tested

### Specificity of the tests

We studied the specificity of the OBTI test using dilutions of semen and saliva and mixtures of these fluids. For semen, respectively saliva, we used 20 µl of material and dilutions of 1, 1/2 and 1/10. For the mixtures, we prepared three semen and saliva mixtures (proportions 1:10, 1:1 and 10:1). These were all deposited on 4N6 FLOQSwabs™. The tests were performed in duplicate and analyzed with both the OBTI and universal buffers. To check that universal buffer did not affect blood detection in mixtures, 20 µl of mixtures of blood and semen as well as blood and saliva (each in the three proportions 1:10, 1:1, and 10:1) and blood, semen, and saliva (in the proportion 1:1:1) was analyzed with this buffer.

The specificity of the PSA test was studied as follows: 20 µl of mixtures of blood and saliva (1:10, 1:1 and 10:1), semen and blood (1:10, 1:1 and 10:1), semen and saliva (1:10, 1:1, and 10:1) and blood, semen and saliva (1:1:1) was deposited on 4N6 FLOQSwabs™ with and without vaginal cells. These items were then analyzed with the universal buffer to detect if this buffer affected semen detection in the mixtures. A female volunteer self-collected vaginal cells by swabbing the vaginal region with 4N6 FLOQSwabs™. The swab was left out to dry before the deposition of the mixtures.

To test for the specificity of the RSID-saliva test, 20 µl of pure semen and blood as well as three mixtures of semen and blood (in proportions 1:10, 1:1, and 10:1) were deposited on 4N6 FLOQSwabs™. These tests were performed in duplicate and analyzed using the universal buffer. Two 4N6 FLOQSwabs™ with vaginal cells collected as described earlier were also tested without adding other fluids. In addition, 20 µl of mixtures of saliva and semen, as well as saliva and blood (in proportions 1:10, 1:1, and 10:1) and blood, semen and saliva (in proportion 1:1:1) was also analyzed with the universal buffer.

### Reading of the test results

The results of the OBTI, PSA, and RSID-saliva tests were read 10 min after the deposition of 100 µl of buffer (120 µl for PSA) onto the respective test strips. When the buffer was red or pink, an extra dilution was applied to avoid the high dose hook effect of the OBTI test [4]. The intensity of the band was compared to an internal calibration scale to determine the test result (see supplementary Fig. 1). The results were declared “positive” when a colored line was observed, “weak positive” in the presence of a very light colored line and “negative” in the absence of a colored line. Christmas tree results were defined as “positive” when more than one spermatozoon was observed, “1 spermatozoon” if only one spermatozoon was observed, “potential spermatozoon” when one or a few cells had characteristics close to those of sperm cells and “negative” when no spermatozoon was observed.

### DNA extractions, DNA quantifications, and STR profiling

The potential impact of the different buffers on the quantity of recovered DNA, and the quality of DNA profiles was studied both for blood and saliva dilutions using a PrepFiler™ automated Forensic DNA Extraction Kit (Applied Biosystems (AB, Foster City, CA) and Hamilton^®^) on AutoLys STAR and ID STARlet platforms as described in [17]. We followed our routine procedure with a PrepFiler™ large volume protocol. DNA was eluted in 50 µl.

For the semen dilutions, DNA was extracted from the swab using the Erase Sperm isolation kit (PTC Laboratories, Columbia, USA) following the manufacturer’s instructions to perform differential lysis and get “non-sperm” and “sperm” fractions [18] with an elution volume of 50 µl for each fraction. DNA was quantified using real time qPCR analysis with the Investigator Quantifiler™ Trio DNA Quantification Kit (Thermo Fisher) on a 7500 Real-Time PCR system instrument as described in [17]. A Wilcoxon Mann–Whitney test was used to test for differences in the distributions of the quantity of recovered DNA with Universal or RSID buffer.

To compare, the limit of detection of the different presumptive tests and the spermatozoa detection with the possibility to obtain a DNA profile, DNA profiling was performed on a selection of dilutions close to and below the limit of detection of the tests. DNA amplification was performed with the AmpFLSTR™ NGM SElect™ PCR Amplification Kit (Applied Biosystems) using a target of 1 ng of template DNA or maximum 10 μl of template DNA, if less than 1 ng was present in the total reaction volume of 25 μl. Amplifications were performed as specified by the manufacturer using 30 PCR-cycles with Veriti thermal cyclers (Thermo Fisher Scientific). Capillary electrophoresis was performed with ABI 3500 XL genetic analyzers (Applied Biosystems) following standard procedures. Peaks of the references were called when they were higher than 70 RFU.

To estimate the quantity of DNA present in the buffer collected for the presumptive tests and therefore not used for STR profiling, we extracted DNA from 50 µl of the buffer available for other tests for each fluid using the same methods. Then, the ratio between the quantities of DNA recovered from this buffer and from the rest of the swab was calculated. This allowed to determine the proportion of DNA “lost” for STR profiling and to ensure that this proportion would not affect the quality of DNA profiles.

## Results

### Blood tests

OBTI tests were positive until dilutions of 1/800 and negative below this limit (Table 1). These results were identical using the OBTI or the universal buffer. Mean DNA quantities recovered after DNA extractions of the swabs with blood dilutions are indicated in Table 1. These quantities were similar using the OBTI or universal buffer (Wilcoxon Mann–Whitney test; *U*-value 556, *p* = 0.88). The proportion of DNA recovered in the universal buffer available for other tests was between 0.0 and 1.4% (Table 1). More than 22 of the 28 alleles of the blood’s donor were detected from profiles obtained from blood dilutions up to 1/4000 (Table 1). Below this dilution, the number of the detected reference alleles was less than 9.

OBTI test results were all negative for the three semen dilutions and for two of the three saliva dilutions (1/2 and 1/10) whereas positive (for OBTI buffer) or weakly positive results (for universal buffer) were obtained for pure saliva (Table 4A). Positive or weakly positive results were also observed for both OBTI and universal buffers for the three mixtures of semen and saliva. Finally, all the mixtures with blood gave positive OBTI test results with the universal buffer (Table 4A).

**Table 4:** Summary of the results of the different specificity tests using various specimens and mixtures: (A) OBTI test using OBTI or universal buffers, (B) PSA using universal buffer on swabs with or without vaginal cells (VC) and (C) RSID-saliva tests

Specimen or mixture tested	Dilution or mixture proportion^2^	A) OBTI test results^1^		B) PSA test results^1^		C) RSID-saliva test^1^
		OBTI buffer	Universal buffer	Universal buffer		Universal buffer
				Without VC	With VC	
Semen	1	-,-	-,-	Sens		+ , +
	1/2	-,-	-,-			NT
	1/10	-,-	-,-			NT
Saliva	1	+ , +	( +),( +)	NT	NT	Sens
	1/2	-,-	-,-	NT	NT	
	1/10	-,-	-,-	NT	NT	
Blood	1	+ , + , +	+ , + , +	NT	NT	-,-
Vaginal swab	NA	NT	NT	NT	NT	+ , +
Semen + saliva	1:10	+ , +	+ , +	+	+	+
	1:1	( +),( +)	( +),( +)	+	+	+
	10:1	( +),( +)	( +),( +)	+	+	+
Blood + semen	1:10	NT	+	+	+	-
	1:1	NT	+	+	+	( +)
	10:1	NT	+	+	+	( +)
Blood + saliva	1:10	NT	+	-	-	+
	1:1	NT	+	-	-	+
	10:1	NT	+	-	-	+
Blood + saliva + semen	1:1:1	NT	+	+	+	+

*NT* not tested, *Sens*. see sensitivity analyses for detailed results

^1^ − Negative, ( +) weak positive, + positive results, ^2^*NA* not applicable

### Semen tests

Sperm cell counts after Christmas tree staining on semen dilutions showed the presence of at least one spermatozoon until semen dilutions of 1/5000 (Table 2). We detected no spermatozoa for the dilutions of 1/10,000. These results were identical using the PSA or universal buffer. The quality and quantity of other important type of cells (epithelial cells, yeast, etc.) that can be observed after a Christmas tree staining seemed also to be unaffected by the use of different buffers (data not shown). The PSA test was also positive until semen dilutions of 1/5000 (Table 2). Again, these results were similar using the PSA or universal buffer although two samples were only scored as weak positives for dilutions of 1/3200 and 1/5000 with the PSA buffer. Mean DNA quantities measured on the non-sperm and sperm fractions recovered after differential DNA extraction of the buccal swabs on which semen dilutions had been deposited are indicated in Table 2. These quantities were similar using the PSA or universal buffer (Wilcoxon Mann–Whitney test; *U*-value 127.5, *p* = 0.55). The proportion of semen DNA recovered in the universal buffer available for other tests calculated from the sperm fraction was between 0.0 and 1.1% (Table 2). Between 6 and 23 of the 31 alleles of the semen donor were called from the DNA profiles obtained for the sperm fraction (Table 2).

PSA test results were all negative in the mixtures without semen and were all positives in the mixtures with semen (Table 4B). These results were similar for all mixtures (i.e., with or without vaginal cells).

### Saliva tests

RSID-saliva tests were positive until saliva dilutions of 1/100, weak positive for saliva dilution of 1/200 and negative below this dilution (Table 3). Mean DNA quantities recovered after the DNA extractions are indicated in Table 3. The proportion of DNA recovered in the universal buffer available for other tests was between 14 and 26% (Table 3). More than 21 of the 28 alleles of the saliva reference were called for the profiles obtained until saliva dilutions of 1/800.

Positive RSID-saliva test were obtained for semen as well as for vaginal swabs but were negative for blood (Table 4C). Weak positive results were observed for two of the three proportions of mixture between semen and blood. Positive RSID-saliva tests were obtained for all mixture proportions with saliva (Table 4C).

The detailed results of all the experiments are available in supplementary Tables 1 to 9.

## Discussion and conclusion

We compared the results obtained for OBTI and PSA tests using our protocol with the universal buffer and with the recommended buffer. We observed no difference in the limit of detection between the different buffers, suggesting that their impact on this parameter is weak or null. The limit of detection for blood with the OBTI test corresponds to a dilution of 1/800. This value is higher than the value reported by [4] but comparable to the limit reported by other studies for methodologies applied to casework [19, 20]. The limit of detection for semen with the PSA test corresponds to a dilution of 1/5000. This value falls between those reported by [8] and [10]. Variation in the preparation of the specimens and in the analytical protocols is a possible explanation for these differences. It is important to note that the choice of the buffer had no visible impact on the sperm cell counts after Christmas tree staining, as the number of sperm cells observed was similar whatever the buffer. At least one sperm cell was observed up to a dilution of 1/5000, which is similar to the limit of detection of the PSA test. Finally, the RSID-saliva test with its recommended buffer showed a limit of detection up to a dilution of 1/200, which roughly corresponds to the value reported by [6].

We were also interested in verifying that the buffer substitution did not decrease the amount of DNA recovered. No effect of the buffer was detected for both automated and differential DNA extractions. To ensure that the quantity of the DNA present in the buffer collected for the presumptive tests was limited, we compared the quantities of DNA recovered from this buffer (and not used for STR profiling) with the quantities of DNA recovered from the swabs. The proportion of DNA present in the buffer represented less than 1.4% for blood and semen, which we consider as negligible. The proportion of DNA present in the buffer for the saliva test ranged from 14 to 26%. It is not clear why a higher proportion of DNA was present in the buffer for the saliva test compared to the blood and semen tests. It is, however, unlikely that this loss could strongly affect the quality of a DNA profile. Interestingly, it was possible to obtain at least partial STR profiles for blood and saliva with dilutions below the limit of detection of the OBTI and RSID-saliva tests. A high variation was observed in the quality of the male STR profiles obtained from semen but at least a partial STR profile was obtained up to the limit of detection of PSA. This suggests that one should obtain a STR profile when the immunochromatographic test is positive for one of these body fluids.

If case information changes during the course of the investigation, a test for the presence of another body fluid may be required. Our protocol allows for this possibility. Preliminary experiments with buffer frozen for at least 12 months (data not shown) have shown a possible small reduction in the sensitivity of the OBTI and RSID-saliva test. Further studies should be conducted to assess the impact of storing buffer for long periods.

The probability of false positive or false negative results depends on various parameters such as the characteristics of the tests and of the biological trace (e.g., size and location of the trace, time since deposition). Semen, vaginal swabs, and mixtures of semen and blood led to unexpected positive or false positive results with the RSID-saliva test. Similar results have been described for semen, vaginal fluid, sweat, breast milk, or feces. These are not surprising as α-amylase is known to be present in these fluids at low concentrations [2]. Our study also showed unexpected positive results with the OBTI test on saliva and mixtures of saliva and semen. Unexpected OBTI results have already been described for saliva, urine, semen, or vaginal fluids and could be explained by the presence of small quantities of blood in these body fluids [4]. Although no false-positive results with the PSA test were detected with saliva and blood mixtures in our study, such false positives have been described for example for urine, vaginal fluids, and rectal swabs [2]. Our results, as well as the fact that the presence of false positives and false negatives strongly varies depending on the studies and test conditions, highlight the importance of taking into account all the information available when interpreting results in a forensic context as recommended in [21]. This can be done by incorporating the various parameters involved in Bayesian Networks as proposed by Wolff and colleagues [12] for saliva tests and Taylor and colleagues [13] for blood tests.

There are two limitations in our study: first, the biological traces were simulated and as such, they do not reflect the complexity of real traces. We are currently using the new protocol on traces from casework; the results conformed to our expectations, although we cannot compare these results to ground truth. Second, the different biological fluids used in this study originated from a limited number of volunteers. This can be problematic as the concentration of the different molecules targeted by the presumptive tests, as well as the DNA concentration within the different biological fluids may fluctuate between persons as well as within a single person over time. This may affect the test sensitivity; however, the effects on specificity and robustness should be limited.

In conclusion, our study indicates that it is possible to use immunochromatographic presumptive tests to detect blood, semen, salivam and DNA on the same eluate (i.e., exact same trace). Our protocol involving the use of the universal buffer gives similar results compared to a situation where each test is performed independently with its own buffer. Furthermore, this does not affect the DNA profile quality. Since the same trace is used for the analysis, the results of the presumptive test(s) and the DNA analyses are representative of the very same biological material.

## Key points

1. Simultaneous detection of different body fluids using the same biological trace is difficult.
2. Three immunochromatographic tests for blood (HEXAGON OBTI), saliva (RSID-saliva) and semen (PSA Semiquant) are combined using the RSID™-universal buffer.
3. Results are similar to those obtained when each test is conducted independently.
4. With the proposed protocol, the results of the presumptive test(s) and the DNA analyses are representative of the very same biological trace.

## Supplementary Information

Below is the link to the electronic supplementary material.

Supplementary file1 (PDF 363 KB)

Supplementary file2 (XLSX 24.5 KB)

## Data Availability

All the data generated or analyzed during this study are included in this published article and its supplementary information files.
