# Supplementary material for: A simplified protocol for the detection of blood, saliva, and semen from a single biological trace using immunochromatographic tests
Source: Forensic Sci Med Pathol. 2022 Feb 16;18(2):141–8. doi: 10.1007/s12024-021-00453-2 (PMC9106612; doi:10.1007/s12024-021-00453-2)
Supplement: Supplementary file 1 — Supplementary file1 (PDF 363 KB) [file 12024_2021_453_MOESM1_ESM.pdf]

Supplementary Figure 1 Internal calibration range for the OBTI (A), PSA (B) and RSID-Saliva (C) tests.

A. OBTI TEST

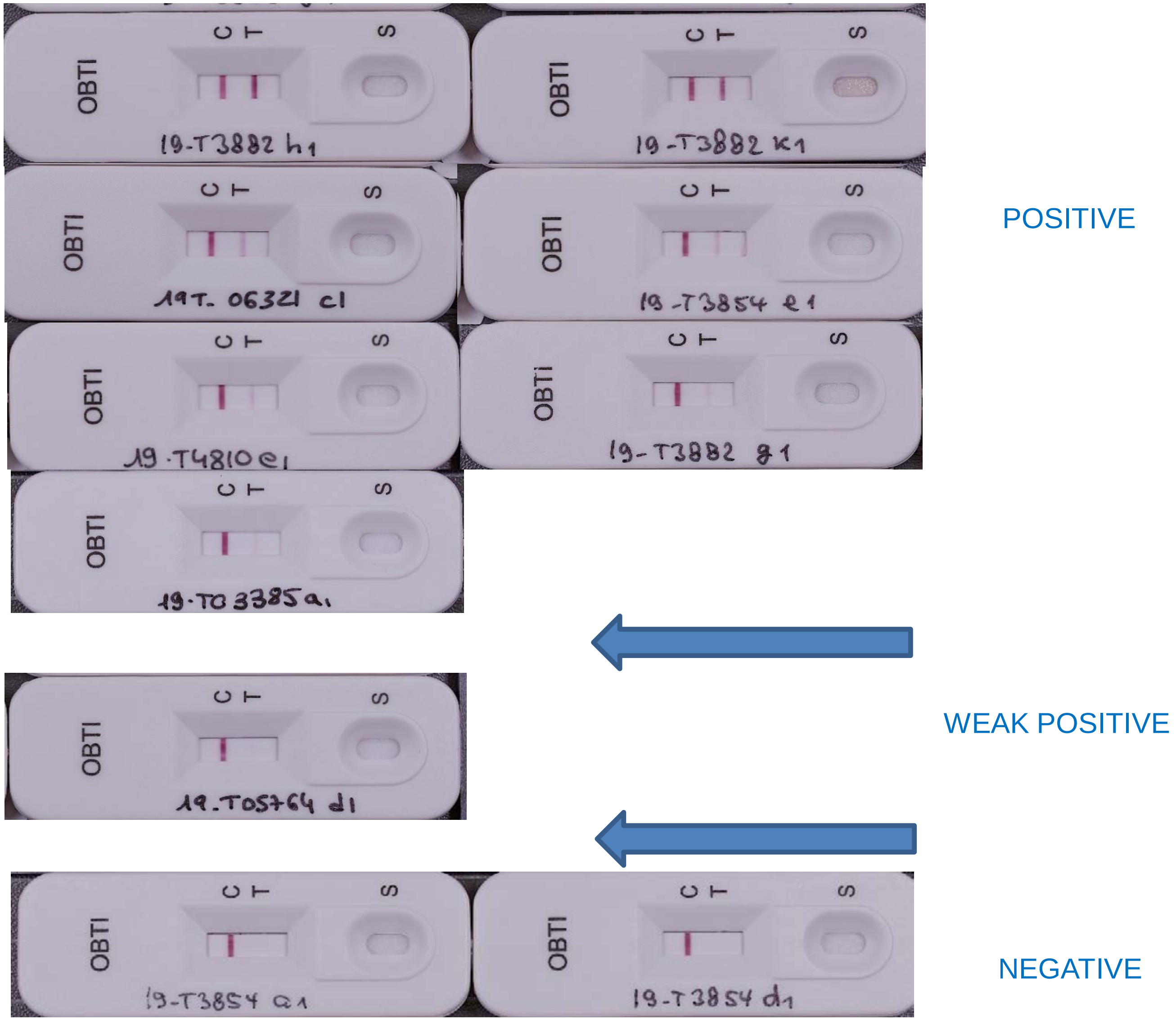

B. PSA TEST

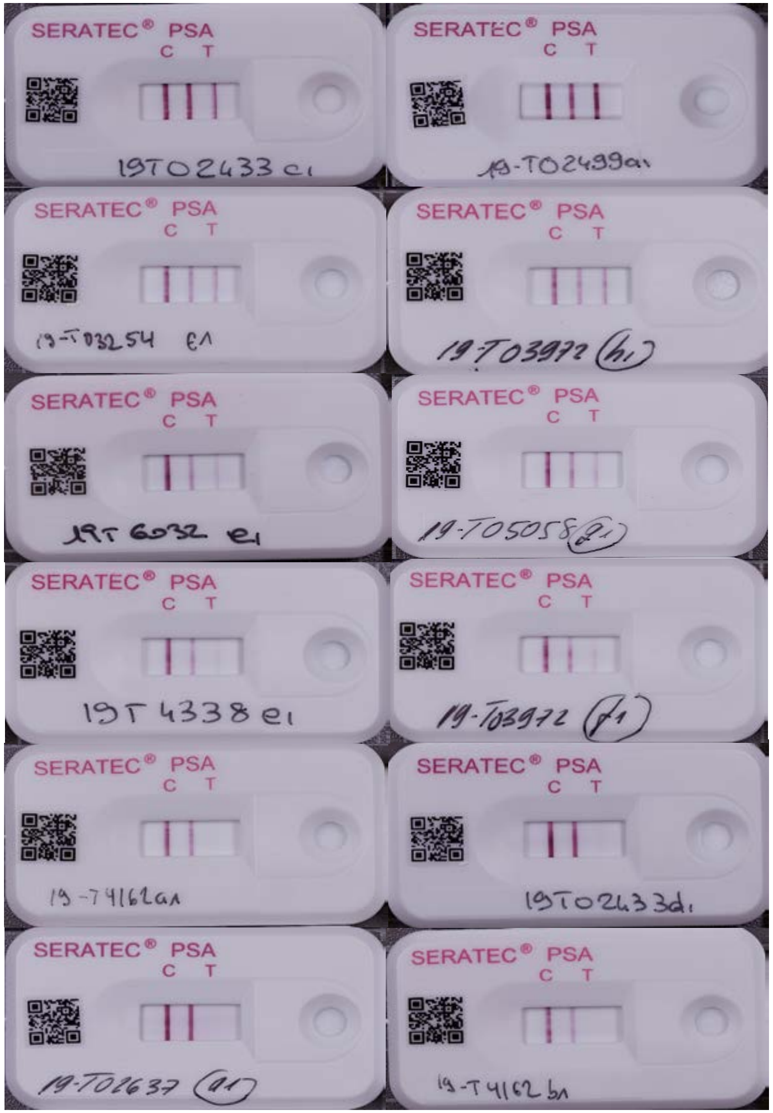

POSITIVE

WEAK POSITIVE

NEGATIVE

C. RSID-Saliva TEST

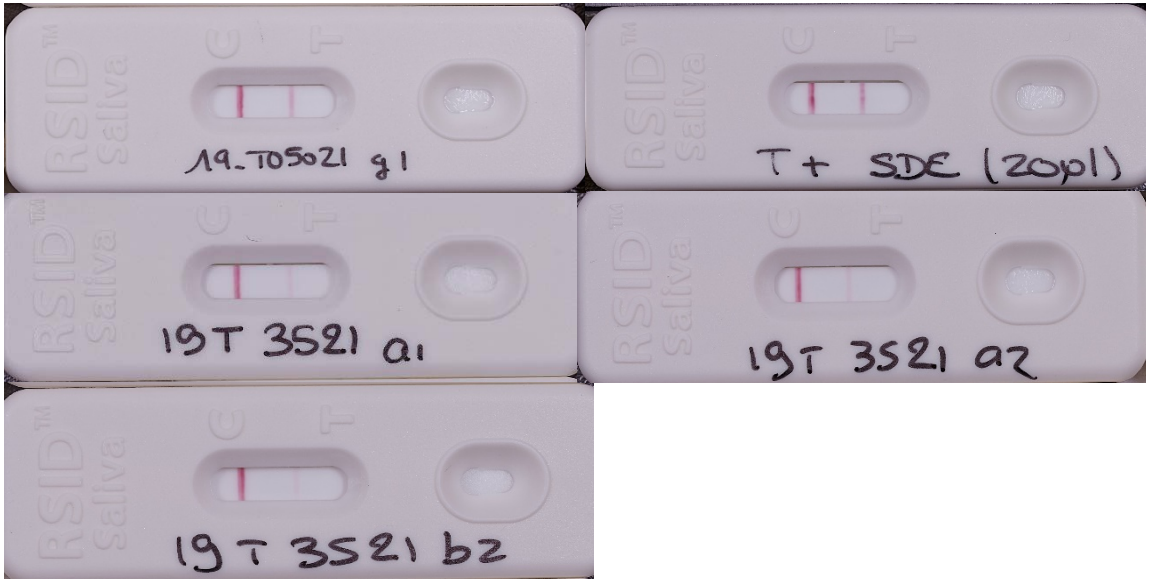

POSITIVE

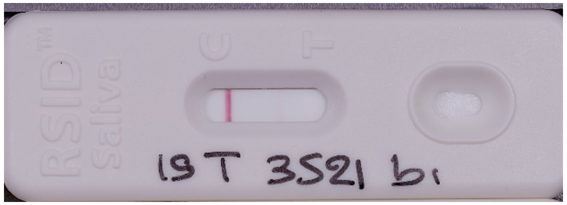

WEAK POSITIVE

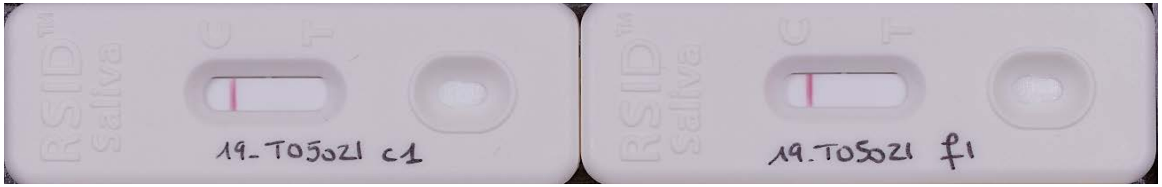

NEGATIVE
